# Use of Metronomic Chemotherapy in Hormone Receptor‐Positive/HER2‐Negative Metastatic Breast Cancer: A Retrospective Multicenter Study

**DOI:** 10.1002/cam4.72325

**Published:** 2026-09-29

**Authors:** Lara Laini, Deborah Cosentini, Marta Laganà, Salvatore Grisanti, Giulia Scartabellati, Gianluca Fogazzi, Salvatore Strazzeri, Vito Amoroso, Greta Schivardi, Rebecca Pedersini, Alfredo Berruti, Antonella Turla

**Affiliations:** ^1^ Department of Medical and Surgical Specialties, Radiological Sciences and Public Health, Medical Oncology University of Brescia, ASST Spedali Civili Brescia Italy; ^2^ Medical Oncology Istituto Clinico Sant'anna Brescia Italy; ^3^ Breast Unit ASST Spedali Civili of Brescia Brescia Italy; ^4^ Medical Oncology ASST Valcamonica Esine Italy

**Keywords:** CDK4/6 inhibitors, hormone receptor‐positive/HER2‐negative breast cancer, metronomic chemotherapy, propensity score matching

## Abstract

**Purpose:**

The optimal chemotherapy strategy after progression on cyclin‐dependent kinase 4/6 inhibitors (CDK4/6i) in hormone receptor‐positive/human epidermal growth factor receptor 2‐negative (HR+/HER2−) metastatic breast cancer remains a matter of controversy. This study evaluated the efficacy of metronomic chemotherapy compared with other post‐CDK4/6i treatment strategies in a real‐world setting.

**Patients and Methods:**

This retrospective multicenter study included patients with HR+/HER2− metastatic breast cancer progressing on CDK4/6i plus endocrine therapy and subsequently treated with systemic therapy. Progression‐free survival (PFS) was the primary endpoint. Secondary endpoints included overall survival (OS), objective response rate (ORR), clinical benefit rate (CBR), and safety. Propensity score matching (1:2) was performed to reduce selection bias.

**Results:**

A total of 177 patients were included, of whom 52 (29.4%) received metronomic chemotherapy. Among treatment strategies, metronomic chemotherapy achieved the longest median PFS (9 months; 95% CI: 5.46–12.53; *p* = 0.003). In multivariable analysis, metronomic chemotherapy was independently associated with prolonged PFS (HR 0.46, 95% CI: 0.30–0.69; *p* < 0.001). Median OS did not significantly differ across treatment groups. ORR was 48.0%, and CBR was 60.0% with metronomic chemotherapy. After propensity score matching, metronomic chemotherapy maintained a significant association with improved PFS (HR 0.49, 95% CI: 0.31–0.78; *p* = 0.003). Toxicities were predominantly Grade 1–2, with no severe adverse events reported in the metronomic cohort.

**Conclusion:**

In this real‐world study, metronomic chemotherapy was associated with improved disease control and a favorable safety profile in patients with HR+/HER2− metastatic breast cancer progressing after CDK4/6i‐based therapy.

**Trial Registration:**

Protocol Number 0037558/23

## 1 Introduction

Over the past decade, the treatment landscape of hormone receptor–positive/human epidermal growth factor receptor 2–negative (HR+/HER2−) metastatic breast cancer (MBC) has been profoundly reshaped by the introduction of cyclin‐dependent kinase 4/6 inhibitors (CDK4/6i) combined with endocrine therapy (ET), which are now established as the standard first‐line treatment approach [1]. However, despite the significant improvement in disease control and survival outcomes, resistance to CDK4/6i inevitably emerges, ultimately leading to disease progression and poorer prognosis [2, 3].

The optimal treatment strategy after progression on CDK4/6i remains undefined. In the absence of direct comparative trials, therapeutic decisions are largely driven by clinical features and tumor biology, resulting in substantial heterogeneity in real‐world practice. More recently, the reassessment of molecular alterations, including PIK3CA, ESR1, AKT, PTEN, and germline BRCA1/2 mutations, has enabled the implementation of biomarker‐driven targeted therapies in selected patients [4, 5, 6, 7, 8, 9, 10]. At the same time, antibody–drug conjugates (ADCs) are increasingly being incorporated into the therapeutic landscape of HR+/HER2− metastatic breast cancer [11, 12]. Nevertheless, chemotherapy continues to play a key role, particularly in patients who are ineligible for, or resistant to, endocrine‐based targeted therapies and ADCs, as well as in those presenting with rapidly progressive disease.

In routine clinical practice, chemotherapy is frequently adopted after CDK4/6i progression, particularly when further endocrine‐based strategies are no longer feasible. Among the available options, metronomic chemotherapy—based on the continuous administration of low‐dose agents—has gained increasing attention because of its favorable tolerability profile and its potential antiangiogenic and immunomodulatory properties [13, 14]. Several studies have reported prolonged clinical benefit with metronomic regimens in metastatic breast cancer [15, 16, 17, 18, 19]. However, whether a metronomic approach may offer advantages over conventional chemotherapy schedules in patients progressing after CDK4/6i remains unclear and is still a matter of debate.

This multicenter retrospective study aimed to evaluate real‐world treatment patterns and outcomes in patients with HR+/HER2− MBC after progression on CDK4/6i plus ET, with a specific focus on the efficacy of metronomic chemotherapy.

## 2 Methods

### 2.1 Study Design and Participants

This retrospective multicenter observational study was conducted to evaluate treatment outcomes after disease progression on CDK4/6i combined with endocrine therapy, with a specific focus on the role of metronomic chemotherapy in patients with HR+/HER2− metastatic breast cancer. Patient data were retrospectively collected from the ASST Spedali Civili di Brescia and the Istituto Clinico Sant'Anna from April 2017 to May 2025. Eligible patients had histologically confirmed HR+ (estrogen and/or progesterone receptor expression ≥ 10%) HER2‐ metastatic or locally advanced unresectable breast cancer. All patients had previously received treatment with a CDK4/6i (palbociclib, ribociclib, or abemaciclib) in combination with ET, including aromatase inhibitors or fulvestrant, and had at least one radiological disease assessment during treatment. Radiologically documented disease progression according to RECIST criteria was required for inclusion.

Patients treated with metronomic chemotherapy after CDK4/6i were compared to a second group with similar clinical characteristics receiving alternative therapies. A propensity score (PS) for receipt of metronomic chemotherapy was estimated using multivariable logistic regression including the following baseline covariates at initiation of post‐CDK4/6i therapy: age (≥ 65 vs. < 65), visceral disease (yes vs. no), prior CDK4/6i duration (< 1 year vs. ≥ 1 year), number of metastatic sites (≥ 3 vs. 1–2), Charlson comorbidity index (≥ 1 vs. 0), and ECOG performance status. Patients were matched using a 1:2 nearest‐neighbor algorithm based on the estimated propensity score.

### 2.2 Statistical Analysis

Categorical variables were reported as absolute frequencies and percentages, and continuous variables were summarized as medians. Survival outcomes were estimated using the Kaplan–Meier method, and median survival times with corresponding 95% confidence intervals (CI) were calculated and compared using the log‐rank test. The association between key clinical variables, treatment type, and survival outcomes was evaluated using Cox proportional hazards regression models in both univariate and multivariable analyses. Results were expressed as hazard ratio (HR) with 95% CI. A *p*‐value < 0.05 was considered statistically significant. Comorbidity burden was assessed using the Charlson Comorbidity Index (CCI), calculated at the time of initiation of treatment following progression on CDK4/6i, and patients were stratified by CCI score (0 vs. ≥ 1) for analysis. All statistical analyses were performed using SPSS version 23.0 (IBM Corp., Armonk, NY, USA).

The primary endpoint was median progression‐free survival (mPFS) associated with metronomic chemotherapy, defined as the time from the start of metronomic chemotherapy to disease progression or last follow‐up. Secondary endpoints included objective response rate (ORR), defined as the proportion of patients achieving complete or partial response, and clinical benefit rate (CBR), defined as complete response, partial response, or stable disease lasting ≥ 6 months. Median overall survival (mOS), defined as the time from treatment initiation to death from any cause or last follow‐up, was also evaluated. In addition, mPFS and mOS were assessed for other therapies administered after progression on CDK4/6 inhibitors. Treatment‐related toxicity was reported according to the Common Terminology Criteria for Adverse Events (CTCAE) Version 5.0.

## 3 Results

### 3.1 Study Population and Clinical Characteristics

Overall, 309 consecutive patients with HR+/HER2− metastatic breast cancer treated with CDK4/6i plus endocrine therapy at the two participating centers were evaluated. Among them, 184 experienced radiologically documented disease progression. Seven patients were subsequently excluded (2 because of receptor conversion and 5 because they were considered unfit for further systemic treatment), resulting in a final study population of 177 patients treated after CDK4/6i progression. The CONSORT diagram (Figure S1) summarizes the patient selection process and reasons for exclusion.

Of these, 134 (75.7%) were treated at ASST Spedali Civili di Brescia and 43 (24.3%) at Istituto Clinico Sant'Anna (Table S1). Palbociclib was most commonly used CDk4/6i (57.5%), followed by ribociclib (25.0%) and abemaciclib (17.5%). CDK4/6i was administered predominantly in the first‐line metastatic setting (74.5%, *n* = 132), with 21.0% (*n* = 37) and 4.5% (*n* = 8) treated in second and later lines. Fulvestrant was the endocrine backbone in 62.7% (*n* = 111), while aromatase inhibitor therapy was prescribed in the remaining 37.3% (*n* = 66). Median CDK4/6i duration was 14 months (range 2–85). Overall, 57.1% (*n* = 101) remained on treatment for at least 12 months, and 42.9% (*n* = 76) discontinued earlier. Best response to CDK4/6i was CR in 5.1% (*n* = 9), PR in 45.2% (*n* = 80), SD in 27.1% (*n* = 48), and PD in 22.6% (*n* = 40).

At the beginning of CDK4/6i treatment i (Table 1), median age was 65 years (range 38–84); ECOG PS was 0 in 75.1% and 1 in 24.9%, and 80.2% had a CCI of 0. Visceral disease was present in 66.7%, most commonly in the liver (44.0%), followed by bone (23.9%) and lung (10.8%). Most patients (71.8%) had 1–2 metastatic sites, and 28.2% had 3 or more.

**TABLE 1:** Baseline patient characteristics after progression to CDK4/6i.

Age, median (range), years	65 (38–84)
ECOG performance status	
0	133 (75.1%)
1	44 (24.9%)
Site of disease progression during CDK4/6i	
Liver	81 (44.0%)
Bone	44 (23.9%)
Lung	20 (10.8%)
Lymph nodes	10 (5.4%)
Skin and soft tissue	7 (3.8%)
Brain	3 (1.6%)
Other visceral sites	19 (10.3%)
Presence of visceral disease	118 (66.7%)
Number of metastatic sites	
1–2	127 (71.8%)
≥ 3	50 (28.2%)
Metastasis biopsy after CDK4/6i progression	25 (13.5%)
Charlson Comorbidity Index (CCI)	
0	142 (80.2%)
1	27 (15.3%)
2	6 (3.4%)
3	2 (1.1%)
CCI (0 vs ≥ 1)	
0	142 (80.2%)
≥ 1	35 (19.8%)
Post‐CDK4/6i treatments	
Chemotherapy (not metronomic schedule)	84 (47.5%)
Taxane‐based	30 (35.8%)
Capecitabine (standard schedule)	29 (34.5%)
Anthracycline‐based	13 (15.4%)
Carboplatin‐based	5 (6.0%)
Vinorelbine	4 (4.8%)
Eribulin	3 (3.5%)
Metronomic chemotherapy	52 (29.4%)
Capecitabine	31 (59.6%)
VEX regimen (vinorelbine‐cyclophosphamide‐capecitabine)	20 (38.4%)
Cyclophosphamide	1 (2.0%)
Endocrine therapy	19 (10.7%)
Fulvestrant	18 (94.7%)
Aromatase inhibitor	1 (5.3%)
Targeted therapy	22 (12.4%)
Everolimus + exemestane	17 (77.2%)
Alpelisib + fulvestrant	4 (18.2%)
Everolimus + elacestrant (ELEVATE trial)	1 (4.6%)

Chemotherapy was the most frequently adopted treatment strategy after CDK4/6i progression (76.9%), including metronomic regimens in 52 patients (29.4%) and conventional chemotherapy in 84 patients (47.5%). Among conventional chemotherapy regimens, taxanes (35.8%) and standard‐dose capecitabine (34.5%) were the most used agents, followed by anthracyclines (15.4%), carboplatin (6.0%), vinorelbine (4.8%), and eribulin (3.5%). Among metronomic regimens, single‐agent capecitabine was the most common (59.6%), followed by the VEX regimen (vinorelbine–cyclophosphamide–capecitabine) (38.4%) and cyclophosphamide alone (2.0%). Endocrine therapy alone was administered in 10.7% (fulvestrant 94.7%), while 12.4% received targeted therapy plus endocrine therapy, mainly everolimus plus exemestane (77.2%).

Among patients with available data, Grade 1, Grade 2, and Grade 3 tumors were observed in 0/36 (0.0%), 6/36 (16.7%), and 30/36 (83.3%) patients in the metronomic chemotherapy group, respectively, compared with 4/77 (5.2%), 32/77 (41.6%), and 41/77 (53.2%) patients treated with other strategies. Grade was unavailable in 16 and 48 patients, respectively. Grade distribution differed significantly between treatment groups (*p* = 0.006), with a higher proportion of Grade 3 tumors in the metronomic chemotherapy group. PIK3CA status was available in 7 patients receiving metronomic chemotherapy and 20 non‐metronomic chemotherapy; mutations were detected in 2/7 (28.6%) and 8/20 (40.0%) patients, respectively (*p* = 0.678). PIK3CA status was not evaluated in 45 and 105 patients, respectively. ESR1 status was available in only 1 patient in the metronomic group and 7 controls; an ESR1 mutation was detected in 1/1 and 4/7 (57.1%) patients, respectively (*p* = 1.000). ESR1 status was not tested in 51 and 118 patients, respectively. Postmenopausal women accounted for 92.3% of patients in the metronomic chemotherapy group and 80.5% of patients submitted to other treatments, with no statistically significant difference between groups (*p* = 0.070).

Among patients treated with metronomic chemotherapy, 35/52 (67.3%) had previously received CDK4/6 inhibitors as first‐line metastatic therapy and 17/52 (32.7%) in the second or later metastatic setting. The corresponding figures were 42/58 (72.4%) and 16/58 (27.6%) for single‐agent chemotherapy, 23/26 (88.5%) and 3/26 (11.5%) for polychemotherapy, 19/19 (100%) and 0/19 (0%) for fulvestrant, and 13/22 (59.1%) and 9/22 (40.9%) for endocrine therapy plus targeted therapy, with no significant differences across treatment groups (*p* = 0.214). All patients treated with fulvestrant alone had previously received CDK4/6 inhibitors as first‐line therapy.

Following progression on the study treatment, the proportion of patients receiving at least one and at least two subsequent treatment lines was comparable between the metronomic chemotherapy and control groups (76.9% vs. 70.4%, *p* = 0.462, and 55.8% vs. 46.4%, *p* = 0.322, respectively). The distribution of subsequent treatment strategies was similar between groups, with chemotherapy representing the most frequently administered first subsequent treatment (61.5% vs. 56.8%, respectively).

### 3.2 PFS After CDK4/6 Inhibitor Progression in the Overall Population

Kaplan–Meier estimates for PFS in the overall population are shown in Figure S2. With a median follow‐up of 42.6 months (95% CI: 31.9–49.0 months), median progression‐free survival (mPFS) was 6.0 months (95% CI: 4.55–7.45). mPFS differed significantly among treatment strategies (heterogeneity test *p* = 0.003), with the longest duration observed in patients receiving metronomic chemotherapy (9 months; 95% CI: 5.46–12.53), compared with 6 months (95% CI: 4.04–7.95) for polychemotherapy, 5 months (95% CI: 2.46–7.53) for single‐agent chemotherapy, 5 months (95% CI: 1.55–8.44) for targeted therapy, and 4 months (95% CI: 3.33–4.66) for endocrine therapy alone (Figure 1). Table S2 compares mPFS (and mOS) among different metronomic regimens.

**FIGURE 1:**
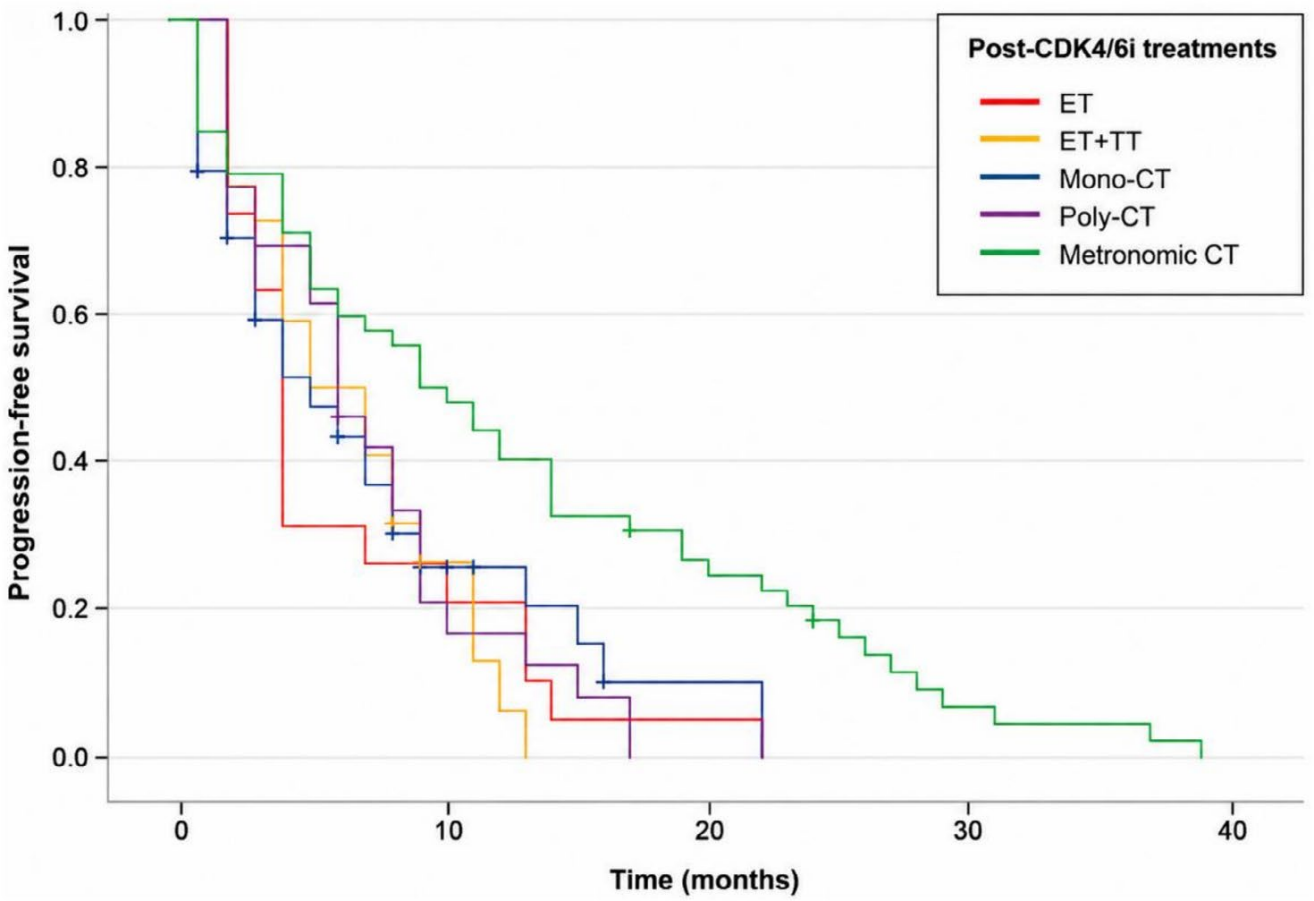
mPFS by type of treatment administered after progression to CDK4/6i.

Univariate and multivariate Cox regression analyses were performed to identify factors associated with PFS (Table 2). In the univariate analysis, metronomic chemotherapy was significantly associated with prolonged PFS (HR 0.48; 95% CI: 0.32–0.70; *p* < 0.001). This association remained significant in the multivariate model, where metronomic chemotherapy independently correlated with a lower risk of disease progression (HR 0.46; 95% CI: 0.30–0.69; *p* < 0.001).

**TABLE 2:** Univariate and multivariate analysis for PFS and OS of clinical and treatment factors.

	PFS						OS					
	Univariate analysis			Multivariate analysis			Univariate analysis			Multivariate analysis		
	HR	95% CI	*p*	HR	95% CI	*p*	HR	95% CI	*p*	HR	95% CI	*p*
Visceral disease												
Yes vs. no	0.93	0.66–1.30	0.673	—	—	—	0.94	0.64–1.4	0.788	—	—	—
aBC *de novo*												
Yes vs. No	1.05	0.69–1.58	0.804	—	—	—	1.05	0.66–1.67	0.825	—	—	—
Age												
< 65 vs. ≥ 65 year	0.88	0.64–1.21	0.440	—	—	—	1.29	0.89–1.87	0.163	—	—	—
CDK4/6i duration												
< 12 vs. ≥ 12 months	1.27	0.92–1.74	0.139	—	—	—	1.76	1.22–2.53	**0.002**	1.75	1.20–2.57	**0.004**
CDK4/6i line												
1st vs. ≥ 2nd	0.99	0.69–1.43	0.988	—	—	—	1.09	0.73–1.63	0.651	—	—	—
CCI												
0 vs. ≥ 1	1.03	0.69–1.51	0.910	—	—	—	1.34	0.86–2.08	0.184	—	—	—
Metronomic CT												
Yes vs. no	0.48	0.32–0.70	< **0.001**	0.46	0.30–0.69	< **0.001**	0.81	0.55–1.19	0.294	—	—	—

*Note:* Bold *p* values if statistically significant.

### 3.3 OS, ORR, CBR After CDK4/6 Inhibitor Progression in the Overall Population

Median OS in the overall population was 22.0 months (95% CI: 19.20–24.80) (Figure S3). No statistically significant differences in OS were observed among the different post‐CDK4/6i treatment strategies (*p* = 0.059) (Figure 2). Median OS with metronomic chemotherapy was 23 months (95% CI: 17.16–28.83), similar to that observed with polychemotherapy (23 months; 95% CI: 17.51–28.48) and only numerically lower than with targeted therapy (29 months; 95% CI: 19.36–38.63). Conversely, single‐agent chemotherapy was associated with the shortest median OS (17 months; 95% CI: 9.43–24.56).

**FIGURE 2:**
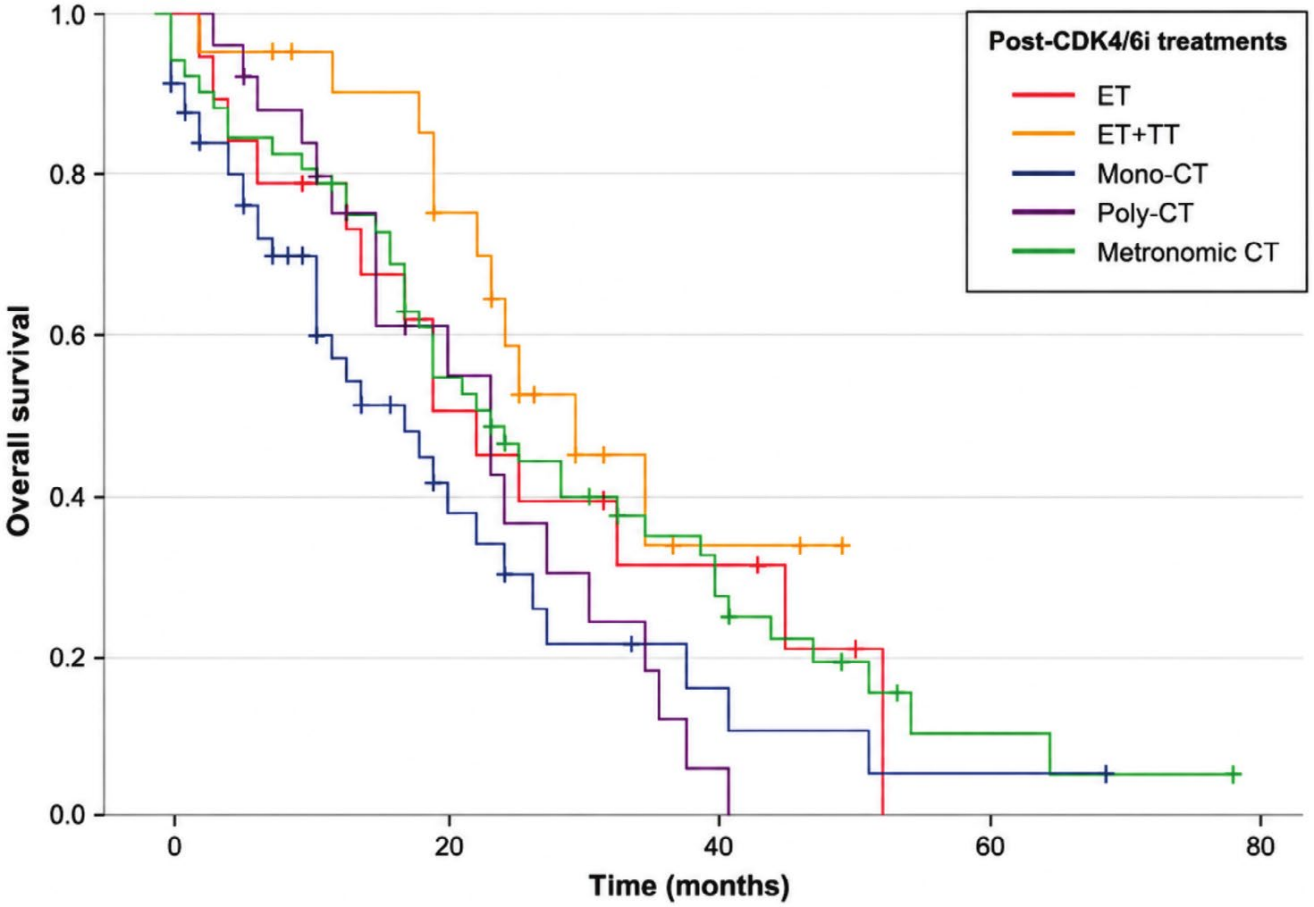
mOS based on the type of treatment administered after progression to CDK4/6i.

In the univariable and multivariable Cox regression analyses for OS (Table 2), a shorter duration of prior CDK4/6i therapy (< 12 vs. ≥ 12 months) was significantly associated with poorer survival both in the univariable analysis (HR 1.76, 95% CI: 1.22–2.53; *p* = 0.002) and in the multivariable model (HR 1.75, 95% CI: 1.20–2.57; *p* = 0.004). In contrast, neither metronomic chemotherapy (HR 0.81, 95% CI: 0.55–1.19; *p* = 0.294) nor the other evaluated clinical variables showed a significant association with OS.

Table S3 summarizes treatment activity according to the type of therapy administered after progression on CDK4/6i. Polychemotherapy achieved the highest ORR (61.5%), followed by metronomic chemotherapy (48.0%), targeted therapy (40.9%), single‐agent chemotherapy (34.5%), and endocrine therapy alone (21.1%), with a statistically significant difference among treatment groups (heterogeneity test: *p* = 0.047). A similar trend was observed for CBR, which was numerically higher with polychemotherapy (65.4%) and metronomic chemotherapy (60.0%) compared with targeted therapy (54.5%), single‐agent chemotherapy (46.6%), and endocrine therapy alone (26.3%). However, differences in CBR across treatment groups did not reach statistical significance (heterogeneity test: *p* = 0.082).

### 3.4 Treatment Outcomes After CDK4/6 Inhibitor Progression After Propensity Score

To avoid potential selection bias, a propensity score analysis was conducted. Propensity score matching yielded a cohort of 120 patients (40 metronomic, 80 controls) (patients' characteristics are reported in Table S4). Within the propensity score‐matched cohort, the proportion of patients with a prior CDK4/6 inhibitor treatment duration of less than 12 months did not differ between the metronomic chemotherapy and not‐metronomic therapy groups among patients who had received CDK4/6 inhibitors as first‐line metastatic therapy (38.5% vs. 42.4%; *p* = 0.814) or in the second or later metastatic setting (57.1% vs. 52.4%; *p* = 1.000). Of the 52 patients treated with metronomic chemotherapy, 51 (98.1%) were treated at ASST Spedali Civili di Brescia and 1 at Istituto Clinico Sant'Anna, reflecting institutional treatment preferences. mPFS was 5 months at ASST Spedali Civili di Brescia and 8 months at Istituto Clinico Sant'Anna (HR for Sant'Anna vs. ASST Spedali Civili 0.85, 95% CI: 0.57–1.27; *p* = 0.433). Similarly, mOS was 22 and 19 months, respectively (HR 1.25, 95% CI: 0.76–2.06; *p* = 0.375). Given the high collinearity between treatment center and treatment allocation, treatment center was not included in the propensity score model. As an additional sensitivity analysis, outcomes were evaluated among patients treated at ASST Spedali Civili di Brescia only. In this subgroup, median PFS was 10 months in patients receiving metronomic chemotherapy and 4 months in those receiving other treatments (HR 0.42, 95% CI: 0.28–0.63; *p* < 0.001), while median OS was 23 and 22 months, respectively (HR 0.83, 95% CI: 0.55–1.25; *p* = 0.372).

After propensity score matching, median PFS remained longer in the metronomic group than in the control group (9.0 vs. 6.0 months) (Figure 3), whereas no significant difference in OS was observed by Kaplan–Meier analysis (23.0 vs. 19.0 months; HR 0.83; 95% CI: 0.56–1.24; *p* = 0.369) (Figure 4). In Cox regression analysis (Table 3), metronomic chemotherapy remained independently associated with improved PFS in both univariable (HR 0.51, 95% CI: 0.34–0.78; *p* = 0.002) and multivariable analyses (HR 0.49, 95% CI: 0.31–0.78; *p* = 0.003) (Table 3). By contrast, no significant association between metronomic chemotherapy and OS was observed in the propensity score–matched cohort (HR 0.75, 95% CI: 0.50–1.11; *p* = 0.151). Among the other evaluated variables, shorter duration of prior CDK4/6i treatment (< 12 vs. ≥ 12 months) was significantly associated with worse OS at both univariable (HR 1.68, 95% CI: 1.05–2.68; *p* = 0.031) and multivariable analyses (HR 1.64, 95% CI: 1.03–2.61; *p* = 0.039). No significant associations with PFS or OS were observed for visceral disease, de novo metastatic disease, age, CDK4/6i treatment line, or Charlson Comorbidity Index (CCI).

**FIGURE 3:**
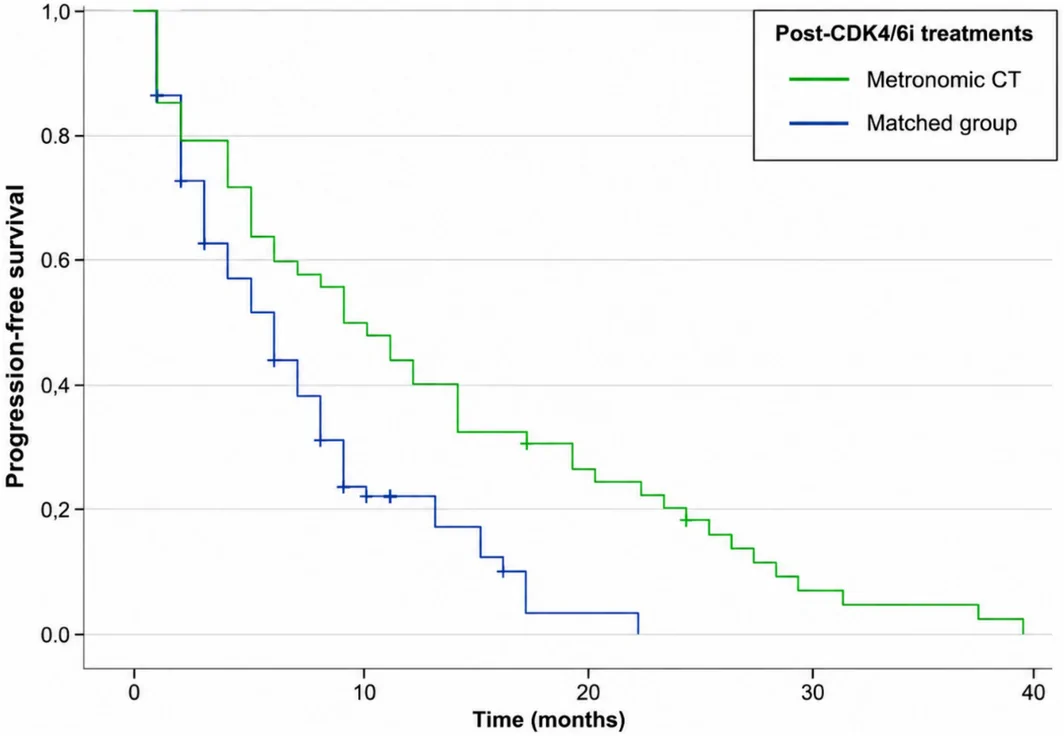
mPFS metronomic vs. non‐metronomic chemotherapy after propensity score matching.

**FIGURE 4:**
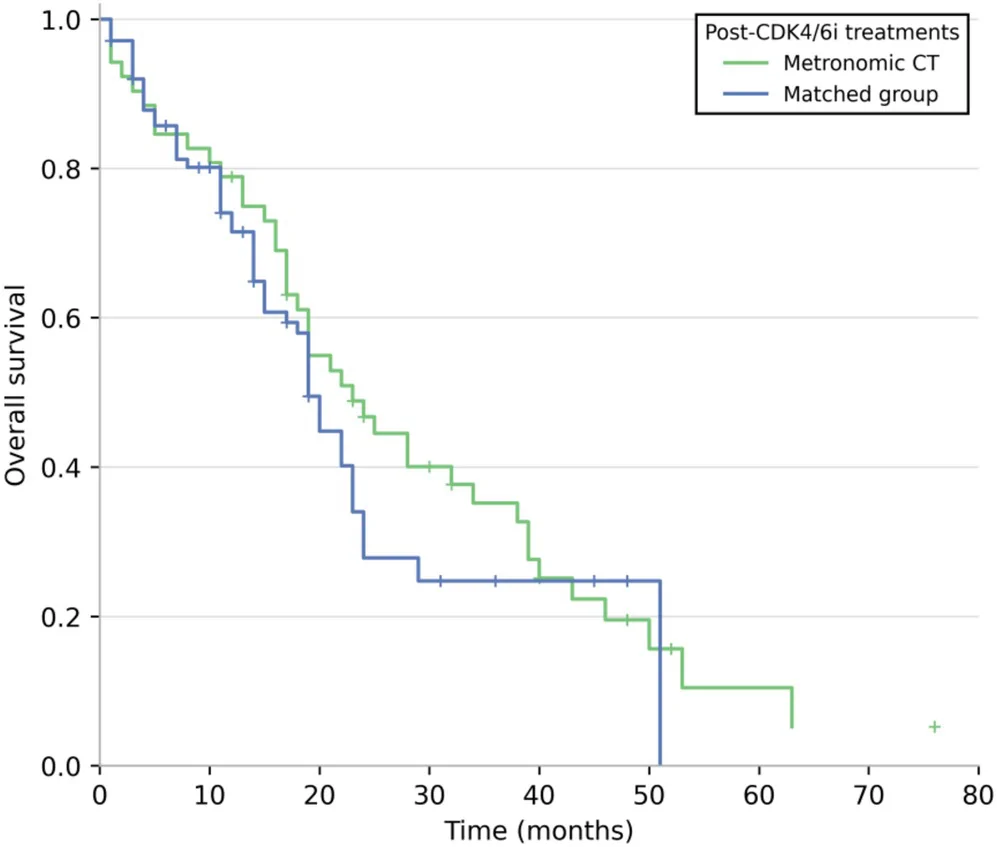
mOS metronomic vs. non‐metronomic chemotherapy after PSM.

**TABLE 3:** Univariate and multivariate analyses of PFS and OS by clinical and treatment factors with metronomic CT post‐CDK4/6i, after propensity score matching.

	PFS						OS					
	Univariate analysis			Multivariate analysis			Univariate analysis			Multivariate analysis		
	HR	95% CI	*p*	HR	95% CI	*p*	HR	95% CI	*p*	HR	95% CI	*p*
Visceral disease												
Yes vs. no	0.88	0.56–1.39	0.584	—	—	—	0.75	0.46–1.24	0.262	—	—	—
aBC *de novo*												
Yes vs. no	0.90	0.46–1.75	0.755	—	—	—	1.06	0.51–2.22	0.879	—	—	—
Age												
< 65 vs. ≥ 65 year	1.05	0.68–1.61	0.827	—	—	—	1.20	0.74–1.95	0.462	—	—	—
CDK4/6i duration												
< 12 vs. ≥ 12 mesi	1.28	0.84–1.96	0.243	—	—	—	1.68	1.05–2.68	**0.031**	1.64	1.03–2.61	**0.039**
CDK4/6i line												
1st vs. ≥ 2nd	0.88	0.53–1.43	0.595	—	—	—	1.04	0.61–1.76	0.888	—	—	—
CCI												
0 vs. ≥ 1	1.18	0.75–1.87	0.470	—	—	—	1.37	0.82–2.28	0.227	—	—	—
Metronomic CT												
Yes vs. no	0.51	0.34–0.78	**0.002**	0.49	0.31–0.78	**0.003**	0.75	0.50–1.11	0.151	—	—	—

*Note:* Bold *p* values if statistically significant.

In the matched population, no significant differences were observed in CBR (57.5% vs. 52.5%; OR 1.22; 95% CI: 0.55–2.75; *p* = 0.624) or ORR (45.0% vs. 41.3%; OR 1.17; 95% CI: 0.51–2.67; *p* = 0.717).

### 3.5 Safety and Toxicity

Adverse events are summarized in Table S5. Non‐chemotherapy treatments were generally associated with mild toxicity. Vasomotor symptoms occurred in 2 patients (10.5%) receiving endocrine therapy, while targeted therapy was associated with mild stomatitis in 27% and one case of severe hyperglycemia. Among non‐metronomic chemotherapy regimens, the most common adverse outcomes were Grade 1–2 diarrhea (26.2%), Grade 1–2 neutropenia (35.7%) and Grade 3–4 neutropenia (8.3%), mucositis (19%), peripheral neuropathy (14%), and hand–foot syndrome (7.1%). In the metronomic chemotherapy group, toxicities were predominantly mild, most commonly diarrhea (15.4%) and mucositis (7.7%), with no severe adverse events reported.

## 4 Discussion

The optimal treatment strategy after progression on CDK4/6i in patients with HR‐positive/HER2‐negative metastatic breast cancer remains an unmet clinical need. In this context, the present paper, specifically focused on the role of metronomic chemotherapy, provides real‐world evidence in a setting where prospective comparative data are still limited.

In our study, the role of metronomic chemotherapy after progression on CDK4/6 inhibitors was explored through two complementary analytical approaches. First, treatment outcomes were evaluated in the overall unselected real‐world population, reflecting the heterogeneity of patients commonly encountered in routine clinical practice. Second, to reduce the potential impact of treatment selection bias and to improve comparability between treatment groups, a propensity score–matched analysis was performed.

Importantly, in both analytical settings metronomic chemotherapy consistently demonstrated a significant advantage in terms of PFS. In the unselected population, metronomic chemotherapy achieved the longest median PFS among all evaluated post‐CDK4/6i treatment strategies and maintained its independent association with reduced risk of progression at multivariable analysis. Notably, this finding was confirmed after propensity score matching, further supporting the robustness and reproducibility of the observed benefit despite the retrospective design of the study. Of note, the metronomic chemotherapy group had a significantly higher proportion of Grade 3 tumors than the control group (83.3% vs. 53.2%), suggesting that the observed PFS advantage was not attributable to a more favorable tumor grade distribution. Nevertheless, this finding should be interpreted cautiously, as tumor grade was unavailable in a substantial proportion of patients and was therefore not included among the variables used for propensity score matching. Although polychemotherapy achieved the highest ORR in the overall cohort, metronomic chemotherapy was associated with the longest mPFS. This apparent discrepancy likely reflects the different therapeutic goals of these approaches: while conventional chemotherapy induces rapid tumor shrinkage, metronomic chemotherapy achieves sustained disease control through continuous low‐dose exposure and its antiangiogenic and immunomodulatory effects. Consistent with this interpretation, the differences in ORR and CBR disappeared after propensity score matching, whereas the mPFS benefit remained unchanged.

By contrast, no statistically significant overall survival advantage emerged across analyses. Although the consistent improvement in PFS indicates a robust benefit in disease control, the impact on OS is inherently more difficult to establish in retrospective real‐world studies of HR‐positive/HER2‐negative metastatic breast cancer, where survival reflects multiple factors beyond the activity of the investigated treatment itself, including intrinsic tumor biology, disease aggressiveness, subsequent lines of therapy, and access to newer therapeutic agents [20, 21, 22, 23, 24]. In the present cohort, the number and overall distribution of post‐progression treatment lines were comparable between treatment groups, making a major imbalance in subsequent treatment exposure an unlikely explanation for the observed OS findings. However, the heterogeneity of subsequent therapies, together with the relatively limited sample size and the prolonged post‐progression survival typical of this disease, may have reduced the ability to detect treatment‐specific differences in OS. Therefore, the absence of a statistically significant OS advantage should be interpreted cautiously and does not necessarily indicate a lack of clinical benefit.

In this context, the consistent prolongation of disease control observed with metronomic chemotherapy acquires particular clinical relevance. Prolonging PFS may allow postponement of more toxic intravenous chemotherapy regimens, maintenance of quality of life, and preservation of patient eligibility for subsequent therapeutic opportunities. This aspect is especially relevant within the evolving therapeutic landscape of HR‐positive/HER2‐negative metastatic breast cancer. According to the most recent ESMO Living Guidelines [1], antibody–drug conjugates are increasingly incorporated into therapeutic algorithms after endocrine‐based strategies. However, in routine clinical practice, access to ADCs frequently remains dependent on prior chemotherapy exposure, particularly in patients with HER2‐low disease, according to current national reimbursement criteria. In this scenario, metronomic chemotherapy may represent a clinically relevant therapeutic strategy, combining meaningful disease control with a favorable toxicity profile and potentially serving as an effective treatment bridge to subsequent ADC‐based therapies.

Beyond efficacy, tolerability represents another major strength emerging from our analysis. Toxicities associated with metronomic chemotherapy were predominantly Grade 1–2, with no severe adverse events reported. In a metastatic setting increasingly characterized by prolonged treatment exposure and therapeutic sequencing, maintaining quality of life and minimizing treatment‐related burden are essential clinical objectives. The favorable safety profile observed in our cohort therefore further supports metronomic chemotherapy as a pragmatic, sustainable, and clinically valuable option after CDK4/6i progression.

## 5 Limitations

This study has limitations that should be acknowledged. First, its retrospective design inherently exposes the analysis to potential selection bias and unmeasured confounding factors. Although a propensity score–matched analysis was performed to improve baseline balance between groups and reduce selection bias, residual confounding cannot be completely excluded. In addition, the relatively limited sample size may have reduced the statistical power of some subgroup analyses, particularly for overall survival, an endpoint that is intrinsically influenced by multiple post‐progression factors, including tumor biology and subsequent lines of therapy. Furthermore, the relatively long study period may have introduced temporal confounding, as treatment algorithms and the availability of biomarker‐driven therapies evolved. Molecular profiling was available only for a limited proportion of patients, particularly for ESR1 and PIK3CA, precluding meaningful biomarker‐stratified outcome analyses and limiting our ability to account for the potential influence of molecular resistance mechanisms on treatment outcomes.

## 6 Conclusion

In this real‐world multicenter analysis, metronomic chemotherapy emerged as a potentially effective and well‐tolerated therapeutic strategy after CDK4/6i progression in patients with HR‐positive/HER2‐negative metastatic breast cancer. The consistent association with prolonged progression‐free survival, observed both in the unselected population and after propensity score adjustment, supports the consistency of the observed clinical benefit. The favorable impact on disease control, together with the excellent tolerability profile, reinforces the clinical relevance of metronomic chemotherapy in the evolving post‐CDK4/6i treatment landscape. Metronomic chemotherapy represents a pragmatic and valuable treatment bridge to subsequent innovative therapies.

## Author Contributions

**Lara Laini:** conceptualization, investigation, data curation, formal analysis, writing – review and editing, writing – original draft. **Deborah Cosentini:** investigation, data curation, writing – review and editing. **Marta Laganà:** investigation, data curation, writing – review and editing. **Salvatore Grisanti:** investigation, resources, writing – review and editing. **Giulia Scartabellati:** investigation, data curation, writing – review and editing. **Gianluca Fogazzi:** investigation, resources, writing – review and editing. **Salvatore Strazzeri:** investigation, resources, writing – review and editing. **Vito Amoroso:** investigation, supervision, writing – review and editing. **Greta Schivardi:** investigation, data curation, writing – review and editing. **Rebecca Pedersini:** conceptualization, methodology, investigation, validation, project administration, supervision, writing – review and editing. **Alfredo Berruti:** conceptualization, methodology, validation, supervision, project administration, writing – original draft, writing – review and editing. **Antonella Turla:** conceptualization, methodology, formal analysis, data curation, investigation, visualization, writing – review and editing, writing – original draft.

## Funding

The authors have nothing to report.

## Ethics Statement

This study was conducted in accordance with the Declaration of Helsinki and was approved by the local Ethics Committees of the participating institutions (approval no. NP 5848).

## Consent

Due to the retrospective nature of the study, informed consent was obtained according to local regulations.

## Conflicts of Interest

The authors declare no conflicts of interest.

## Supporting information


**Table S1:** Patient characteristics at diagnosis, initiation of CDK4/6i treatment, and initiation of post‐CDK4/6i treatment.
**Table S2:** Survival outcomes according to individual metronomic chemotherapy regimens.
**Table S3:** Best response, CBR and ORR in relation to the type of treatment.
**Table S4:** Baseline characteristics after propensity score matching.
**Table S5:** Adverse events associated with post CDK4/6i treatments.

**Figure S1:** Consort diagram of enrolled patients.
**Figure S2:** Median progression‐free survival (mPFS) post CDK4/6i.
**Figure S3:** Median overall survival (mOS) post CDK4/6i.

## Data Availability

The data that support the findings of this study are available from the corresponding author upon reasonable request.

## References

[1] A. Gennari , F. André , C. H. Barrios , et al., “ESMO Clinical Practice Guideline for the Diagnosis, Staging and Treatment of Patients With Metastatic Breast Cancer,” Annals of Oncology 32, no. 12 (2021): 1475–1495, 10.1016/j.annonc.2021.09.019. 34678411

[2] M. R. Lloyd , L. M. Spring , A. Bardia , and S. A. Wander , “Mechanisms of Resistance to CDK4/6 Blockade in Advanced Hormone Receptor—Positive, HER2‐Negative Breast Cancer and Emerging Therapeutic Opportunities,” Clinical Cancer Research 28 (2022): 821–830, 10.1158/1078-0432.CCR-21-2947. 34725098

[3] D. Basile , L. Gerratana , C. Corvaja , et al., “First‐ and Second‐Line Treatment Strategies for Hormone‐Receptor (HR)‐Positive HER2‐Negative Metastatic Breast Cancer: A Real‐World Study,” Breast 57 (2021): 104–112. 10.1016/j.breast.2021.02.015 PMC8053791 33812267

[4] H. J. Burstein , A. DeMichele , L. Fallowfield , et al., “Endocrine and Targeted Therapy for Hormone Receptor‐Positive, Human Epidermal Growth Factor Receptor 2–Negative Metastatic Breast Cancer‐Capivasertib‐Fulvestrant: ASCO Rapid Recommendation Update,” Journal of Clinical Oncology 42, no. 12 (2024): 1450–1453, 10.1200/JCO.24.00248. 38478799

[5] H. S. Rugo , F. Lerebours , E. Ciruelos , et al., “Alpelisib Plus Fulvestrant in PIK3CA‐Mutated, Hormone Receptor‐Positive Advanced Breast Cancer After a CDK4/6 Inhibitor (BYLieve): One Cohort of a Phase 2, Multicentre, Open‐Label, Non‐Comparative Study,” Lancet Oncology 22, no. 4 (2021): 489–498, 10.1016/S1470-2045(21)00034-6. 33794206

[6] N. C. Turner , M. Oliveira , S. J. Howell , et al., “Capivasertib in Hormone Receptor–Positive Advanced Breast Cancer,” New England Journal of Medicine 388, no. 22 (2023): 2058–2070, 10.1056/NEJMoa2214131. PMC11335038 37256976

[7] J. A. Beaver and B. H. Park , “The BOLERO‐2 Trial: The Addition of Everolimus to Exemestane in the Treatment of Postmenopausal Hormone Receptor‐Positive Advanced Breast Cancer,” Future Oncology 8, no. 6 (2012): 651–657, 10.2217/fon.12.49. PMC3466807 22764762

[8] F.‐C. Bidard , V. G. Kaklamani , P. Neven , et al., “Elacestrant (Oral Selective Estrogen Receptor Degrader) Versus Standard Endocrine Therapy for Estrogen Receptor–Positive, Human Epidermal Growth Factor Receptor 2–Negative Advanced Breast Cancer: Results From the Randomized Phase III EMERALD Trial,” Journal of Clinical Oncology 40 (2022): 3246–3256. 10.1200/JCO.22.00338 PMC9553388 35584336

[9] M. E. Robson , N. Tung , P. Conte , et al., “OlympiAD Final Overall Survival and Tolerability Results: Olaparib Versus Chemotherapy Treatment of Physician's Choice in Patients With a Germline BRCA Mutation and HER2‐Negative Metastatic Breast Cancer,” Annals of Oncology 30, no. 4 (2019): 558–566, 10.1093/annonc/mdz012. PMC6503629 30689707

[10] J. K. Litton , S. A. Hurvitz , L. A. Mina , et al., “Talazoparib Versus Chemotherapy in Patients With Germline BRCA1/2‐Mutated HER2‐Negative Advanced Breast Cancer: Final Overall Survival Results From the EMBRACA Trial,” Annals of Oncology 31, no. 11 (2020): 1526–1535, 10.1016/j.annonc.2020.08.2098. PMC10649377 32828825

[11] G. Curigliano , X. Hu , R. A. Dent , et al., “Trastuzumab Deruxtecan (T‐DXd) vs Physician's Choice of Chemotherapy (TPC) in Patients (Pts) With Hormone Receptor–Positive (HR+), Human Epidermal Growth Factor Receptor 2 (HER2)‐Low or HER2‐Ultralow Metastatic Breast Cancer (mBC) With Prior Endocrine Therapy (ET): Primary Results From DESTINY‐Breast06 (DB‐06),” Journal of Clinical Oncology 42, no. 17 (2024): LBA1000, 10.1200/JCO.2024.42.17_suppl.LBA1000.

[12] H. S. Rugo , A. Bardia , F. Marm'e , et al., “Overall Survival With Sacituzumab Govitecan in Hormone Receptor‐Positive and Human Epidermal Growth Factor Receptor 2‐Negative Metastatic Breast Cancer (TROPiCS‐02): A Randomised, Open‐ Label, Multicentre, Phase 3 Trial,” Lancet 402, no. 10411 (2023): 1423–1433, 10.1016/S0140-6736(23)01245-X. 37633306

[13] D. Hanahan , G. Bergers , and E. Bergsland , “Less Is More, Regularly: Metronomic Dosing of Cytotoxic Drugs Can Target Tumor Angiogenesis in Mice,” Journal of Clinical Investigation 105 (2000): 1045–1047. 10.1172/JCI9872 PMC300842 10772648

[14] M. E. Lutsiak , R. T. Semnani , D. P. Roberto , et al., “Inhibition of CD4+25+ T Regulatory Cell Function Implicated in Enhanced Immune Response by Low‐Dose Cyclophosphamide,” Blood 105 (2005): 2862–2868. 10.1182/blood-2004-06-2410 15591121

[15] M. R. Stockler , V. J. Harvey , P. A. Francis , et al., “Capecitabine Versus Classical Cyclophosphamide, Methotrexate, and Fluorouracil as First‐Line Chemotherapy for Advanced Breast Cancer,” Journal of Clinical Oncology 29 (2011): 4498–4504. 10.1200/JCO.2010.33.9101 22025143

[16] P. Fedele , A. Marino , L. Orlando , et al., “Efficacy and Safety of Low‐Dose Metronomic Chemotherapy With Capecitabine in Heavily Pretreated Patients With Metastatic Breast Cancer,” European Journal of Cancer 48 (2012): 24–29. 10.1016/j.ejca.2011.06.040 21775129

[17] R. Addeo , A. Sgambato , G. Cennamo , et al., “Low‐Dose Metronomic Oral Administration of Vinorelbine in the First‐Line Treatment of Elderly Patients With Metastatic Breast Cancer,” Clinical Breast Cancer 10 (2010): 301–306. 10.3816/CBC.2010.n.039 20705563

[18] E. Montagna , A. Palazzo , P. Maisonneuve , et al., “Safety and Efficacy Study of Metronomic Vinorelbine, Cyclophosphamide Plus Capecitabine in Metastatic Breast Cancer: A Phase II Trial,” Cancer Letters 400 (2017): 276–281. 10.1016/j.canlet.2017.01.027 28131905

[19] E. Munzone , M. M. Regan , S. Cinieri , et al., “Efficacy of Metronomic Oral Vinorelbine, Cyclophosphamide, and Capecitabine vs Weekly Intravenous Paclitaxel in Patients With Estrogen Receptor‐Positive, ERBB2‐Negative Metastatic Breast Cancer: Final Results From the Phase 2 METEORA‐II Randomized Clinical Trial,” JAMA Oncology 9 (2023): 1267–1272. 10.1001/jamaoncol.2023.2150 PMC10346502 37440239

[20] C. Karacin , B. Oksuzoglu , A. Demirci , et al., “Efficacy of Subsequent Treatments in Patients With Hormone‐Positive Advanced Breast Cancer Who Had Disease Progression Under CDK 4/6 Inhibitor Therapy,” BMC Cancer 23 (2023): 136. 10.1186/s12885-023-10609-8 PMC9912535 36765293

[21] J. M. Martin , E. A. Handorf , A. J. Montero , and L. J. Goldstein , “Systemic Therapies Following Progression on First‐Line CDK4/6‐Inhibitor Treatment: Analysis of Real‐World Data,” Oncologist 27, no. 6 (2022): 441–446. 10.1093/oncolo/oyac075 PMC9177121 35552450

[22] P. P. M. Berton Giachetti , S. Morganti , S. Gandini , et al., “Survival Following CDK4/6 Inhibitor Therapy for Hormone Receptor‐Positive, ERBB2‐Negative Metastatic Breast Cancer,” JAMA Network Open 8, no. 2 (2025): e2461067. 10.1001/jamanetworkopen.2024.61067 PMC11846014 39982725

[23] L. Bender , C. Pflumio , P. Trensz , et al., “Capecitabine Efficacy After Cycline‐Dependent‐Kinase 4/6 Inhibitor Plus Endocrine Therapy in Metastatic Hormone Receptor‐Positive Breast Cancer,” Cancer Treatment and Research Communications 36 (2023): 100738. 10.1016/j.ctarc.2023.100738 37390791

[24] A. Bardia , X. Hu , R. Dent , DESTINY‐Breast06 trial investigators , et al., “Trastuzumab Deruxtecan After Endocrine Therapy in Metastatic Breast Cancer,” New England Journal of Medicine 391, no. 12 (2024): 1105–1119, 10.1056/NEJMoa2407086. 39282896

