# Supplementary material for: Use of Metronomic Chemotherapy in Hormone Receptor‐Positive/HER2‐Negative Metastatic Breast Cancer: A Retrospective Multicenter Study
Source: Cancer Med. 2026 Sep 29;15(10):e72325. doi: 10.1002/cam4.72325 (PMC13624525; doi:10.1002/cam4.72325)
Supplement: Supplementary file 1 [file CAM4-15-e72325-s001.docx]

**Supplementary figures**

**Figure S1 Consort diagram of enrolled patients.**

Patients treated with CDK4/6i
(n=309)

CDK4/6i ongoing
(n=125)

Disease progression on CDK4/6i
(n=184)

HER2+ or TN at biopsy
(n=2)

Clinical conditions not allowing further treatment
(n=5)

Patients underwent post-CDK4/6i treatments
(n=177)

**Figure S2.** Median progression-free survival (mPFS) post CDK4/6i.

**Figure S3.** Median overall survival (mOS) post CDK4/6i.
