# Supplementary material for: Use of Metronomic Chemotherapy in Hormone Receptor‐Positive/HER2‐Negative Metastatic Breast Cancer: A Retrospective Multicenter Study
Source: Cancer Med. 2026 Sep 29;15(10):e72325. doi: 10.1002/cam4.72325 (PMC13624525; doi:10.1002/cam4.72325)
Supplement: Supplementary file 2 [file CAM4-15-e72325-s002.docx]

**Supplementary tables:**

**Supplementary Table 1** Patient characteristics at diagnosis, initiation of CDK4/6i treatment, and initiation of post-CDK4/6i treatment.

| **Patient characteristics** | **n=177 (%)** |
| --- | --- |
| ICS | 43 (24.3%) |
| SC | 134 (75.7%) |
| Age at diagnosis, median (range), years | 54 (27-79) |
| Sex  Male  Female | 2 (1.1%)  175 (98.9%) |
| Hystological subtype |  |
| Ductal | 130 (73.5%) |
| Lobular | 38 (21.4%) |
| Mixed | 9 (5.1%) |
| aBC *de novo* | 32 (18.1%) |
| Neoadjuvant chemotherapy | 16 (9.0%) |
| Adjuvant chemotherapy | 87 (49.0%) |
| Adjuvant endocrine therapy | 132 (74.6%) |
| Age at initiation of CDK4/6i treatment, median (range) | 63 (37-83) |
| Treatment line with CDK4/6i |  |
| First | 132 (74.5%) |
| Second | 37 (21.0%) |
| ≥ Third | 8 (4.5%) |
| Type of CDK 4/6i |  |
| Palbociclib | 102 (57.5%) |
| Ribociclib | 44 (25.0%) |
| Abemaciclib | 31 (17.5%) |
| Duration of CDK4/6i therapy  ≥ 12 months  < 12 months | 101 (57.1%)  76 (42.9%) |
| Duration of CDK4/6i therapy  Median (range), months | 14 (2-85) |
| ET in combination with CDK4/6i |  |
| AI | 66 (37.3%) |
| Fulvestrant | 111 (62.7%) |
| Better response to CDK4/6i treatment |  |
| CR | 9 (5.1%) |
| PR | 80 (45.2%) |
| SD | 48 (27.1%) |
| PD | 40 (22.6%) |

**Supplementary Table 2.** Survival outcomes according to individual metronomic chemotherapy regimens

| **Metronomic regimen** | **n** | **Median PFS (months)** | **Median OS (months)** | **PFS HR (95% CI)** | **PFS p value** | **OS HR (95% CI)** | **OS p value** |
| --- | --- | --- | --- | --- | --- | --- | --- |
| **Metronomic capecitabine** | 31 | 9 | 22 | 0.76 (0.43–1.37) | 0.365 | 0.79 (0.42–1.49) | 0.458 |
| **VEX (vinorelbine–cyclophosphamide–capecitabine)** | 20 | 14 | 23 |  |  |  |  |
| **Cyclophosphamide monotherapy** | 1 | 1 | 2 | Not estimable | — | Not estimable | — |

**Supplementary Table 3.** Best response, CBR and ORR in relation to the type of treatment.

|  | **Endocrine therapy** | **Target therapy** | **Single agent-**  **chemotherapy** | **Poli-**  **chemotherapy** | **Metronomic** | ***p*** |
| --- | --- | --- | --- | --- | --- | --- |
| **CBR** |  |  |  |  |  |  |
| Yes | 5 (26.3%) | 12 (54.5%) | 27 (46.6%) | 17 (65.4%) | 31 (60.0%) |  |
| No | 14 (73.7%) | 10 (45.5%) | 31 (53.4%) | 9 (34.6%) | 21 (40.0%) | 0.082 |
| **ORR** |  |  |  |  |  |  |
| Yes | 4 (21.1%) | 9 (40.9%) | 20 (34.5%) | 16 (61.5%) | 25 (48.0%) |  |
| No | 15 (78.9%) | 13 (59.1%) | 38 (65.5%) | 10 (38.5%) | 27 (52.0%) | **0.047** |
| **Best response** | |  |  |  |  |  |
| CR | 0 (0%) | 0 (0%) | 2 (3.4%) | 0 (0%) | 2 (3.8%) |  |
| PR | 4 (21.1%) | 9 (40.9%) | 18 (31.0%) | 16 (61.5%) | 23 (44.2%) | 0.223 |
| SD | 2 (10.5%) | 3 (13.6%) | 12 (20.7%) | 3 (11.5%) | 6 (11.5%) |  |
| PD | 13 (68.4%) | 10 (45.5%) | 26 (44.8%) | 7 (26.9%) | 21 (40.4%) |  |

**Supplementary Table 4. Baseline characteristics after propensity score matching**

| **Characteristic** | **Metronomic chemotherapy (n=40)** | **Control group (n=80)** | **p value** |
| --- | --- | --- | --- |
| Age ≥65 years | 21 (52.5) | 42 (52.5) | 1.000 |
| Visceral disease | 26 (65.0) | 57 (71.3) | 0.48 |
| Prior CDK4/6 inhibitor duration <1 year | 18 (45.0) | 36 (45.0) | 1.000 |
| ≥3 metastatic sites | 12 (30.0) | 29 (36.3) | 0.50 |
| Charlson Comorbidity Index ≥1 | 8 (20.0) | 11 (13.8) | 0.38 |
| ECOG performance status 1 | 10 (25.0) | 25 (31.3) | 0.47 |

**Supplementary Table 5.** *Adverse events associated with post CDK4/6i treatments.*

| **Post CDK4/6i treatment** | **n=177** | **adverse events** | **G1–G2 n (%)** | **G3–G4 n (%)** |
| --- | --- | --- | --- | --- |
| endocrine therapy | 19 (10.7%) | vasomotor symptoms | 2 (10.5%) | – |
| targeted therapy | 22 (12.4%) | stomatitis | 6 (27.0%) | – |
|  |  | hyperglycemia | – | 1 (4.5%) |
| non-metronomic chemotherapy | 84 (47.5%) | Diarrhea | 22 (26.2%) | – |
|  |  | Hand-foot syndrome | 6 (7.1%) | – |
|  |  | Neutropenia | 30 (35.7%) | 7 (8.3%) |
|  |  | Nucositis | 16 (19.0%) | 2 (2.4%) |
|  |  | Neuropathy | 12 (14.0%) | – |
| metronomic chemotherapy | 52 (29.4%) | Diarrhea | 8 (15.4%) | – |
|  |  |  |  |  |
|  |  | Mucositis | 4 (7.7%) | – |
